# 3D trunk orientation measured using inertial measurement units during anatomical and dynamic sports motions

**DOI:** 10.1111/sms.13851

**Published:** 2020-12-07

**Authors:** Niels P. Brouwer, Ted Yeung, Maarten F. Bobbert, Thor F. Besier

**Affiliations:** ^1^ Department of Human Movement Sciences, Faculty of Behavioural and Movement Sciences Vrije Universiteit Amsterdam, Amsterdam Movement Sciences Amsterdam The Netherlands; ^2^ Auckland Bioengineering Institute University of Auckland Auckland New Zealand; ^3^ Department of Engineering Science University of Auckland Auckland New Zealand

**Keywords:** accuracy, IMU, three‐dimensional, trunk rotation

## Abstract

Trunk motion is related to the performance and risk of injuries during dynamic sports motions. Optical motion capture is traditionally used to measure trunk motion during dynamic sports motions, but these systems are typically constrained to a laboratory environment. Inertial measurement units (IMUs) might provide a suitable alternative for measuring the trunk orientation during dynamic sports motions. The objective of the present study was to assess the accuracy of the three‐dimensional trunk orientation measured using IMUs during dynamic sports motions and isolated anatomical trunk motions. The motions were recorded with two IMUs and an optical motion capture system (gold standard). Ten participants performed a total of 71 sports motions (19 golf swings, 15 one‐handed ball throws, 19 tennis serves, and 18 baseball swings) and 125 anatomical trunk motions (42, 41, and 42 trials of lateral flexion, axial rotation, and flexion/extension, respectively). The root‐mean‐square differences between the IMU‐ and optical motion capture‐based trunk angles were less than 5 degrees, and the similarity between the methods was on average across all trials “very good” to “excellent” (R ≥ 0.85; *R* ^2^ ≥ 0.80). Across the dynamic sports motions, even higher measures of similarity were found (*R* ≥ 0.90; *R* ^2^ ≥ 0.82). When aligned to the relevant segment, the current IMUs are a promising alternative to optical motion capture and previous presented IMU‐based systems for the field‐based measurement of the three‐dimensional trunk orientation during dynamic sports motions and the anatomical trunk motions.

## 1 INTRODUCTION

Trunk motion contributes to the technique of almost all sports motions. In this context, trunk motion refers to the motion of the segment incorporating the thorax and abdomen relative to the pelvis.^1^ The trunk is essential in maintaining stability of the human body^1^, ^2^ and contains large muscle groups that are responsible for generating upper and lower limb motion. The anatomical trunk motions flexion/extension, lateral flexion, and axial rotation (trunk motion in the sagittal, frontal, and transverse plane, respectively) contribute to important performance parameters, such as ball release velocity during a handball throw,^3^ soccer instep kick,^4^ field hockey drag flick,^5^ golf swing,^6^ tennis serve,^7^ and cricket fast bowling^8^ or power output during flat‐water kayak paddling.^9^ During dynamic sports motions, trunk motion is also associated with the risk of lower limb, upper limb, and spinal injuries. For example, contralateral axial rotation and lateral flexion of the trunk during sidestep cutting tasks lead to increased knee joint moments,^10^, ^11^ and consequently a higher risk of knee ligament injury.^12^ The timing of axial rotation during a baseball pitch has been found to affect the risk of shoulder overuse injury.^13^ In cricket fast bowlers, the magnitude of trunk axial rotation is associated with soft tissue injuries and fractures in the spine.^8^ Measuring trunk motion during dynamic sports motions is critical to improve our understanding of the role of trunk motion in both sports performance and sports injury.

Trunk motion during dynamic sports motions has been measured traditionally using optical motion capture. Although it is considered the “gold standard” method for kinematic measurement, optical motion capture suffers limitations for measuring segmental and joint motion, particularly in sports. Limitations include high cost of the camera equipment, long set‐up and processing times, the need to track multiple markers, and marker occlusion. Most importantly, optical motion capture is typically constrained to a laboratory environment. Small, wearable inertial measurement units (IMUs) might provide a suitable alternative to optical motion capture for the field‐based measurement of trunk motion during dynamic sports motions.

An IMU typically consists of a three‐axis accelerometer, gyroscope, and magnetometer to measure the three‐dimensional linear acceleration, angular velocity, and earth magnetic field, respectively. These data can be combined using a “data fusion” algorithm to estimate the three‐dimensional orientation of the IMU with respect to a global coordinate frame.^14^ When one IMU is attached to the trunk and a second IMU is attached to the pelvis, the three‐dimensional orientation of the trunk relative to the pelvis can be measured. However, several technical limitations are associated with obtaining orientation measurements from IMUs. The integrated gyroscope signal drifts over time due to gyroscope measurement errors and consequently the accuracy of the IMU‐based orientation estimation could decrease over time. Data fusion algorithms can correct the gyroscope drift based on the accelerometer and magnetometer data.^14^ However, the earth magnetic field can be distorted due to nearby metal objects and, during high accelerations, the gravity vector may become indistinguishable from linear accelerations. This could result in a limited correction for gyroscope drift and consequently in a decreased accuracy of the IMU orientation estimation. Most importantly, calibration is required to convert changes in orientation of the IMU with respect to the global earth coordinate frame into anatomical segment or joint motion. Considering these limitations, the validity of the three‐dimensional trunk orientation (ie, the orientation of the trunk in its three anatomical planes) estimated with body‐worn IMUs should be investigated.

The accuracy of the three‐dimensional trunk orientation measured using IMUs has been evaluated previously for clinical tasks, such as box lifting,^15^, ^16^, ^17^, ^18^ isolated anatomical trunk motions,^19^ and the timed‐up‐and‐go walking assessment.^20^ These studies compared the trunk orientation, either relative to the pelvis (using two IMUs) or a global coordinate system (using a single IMU), measured during clinical tasks using wired IMU’s with the optical motion capture‐based trunk orientation (“gold standard”). Root‐mean‐square errors were typically within 5º and Pearson’s correlation coefficient values generally higher than 0.80, indicating that the trunk orientation can accurately be determined using these IMU systems during clinical tasks.^15^, ^16^, ^17^, ^18^, ^19^, ^20^ However, IMU systems comprised of body‐worn wired IMUs, batteries, and logging devices may hamper and limit movement, especially during dynamic sports motions. A few studies evaluated the accuracy of trunk orientation relative to the pelvis measured using wireless IMUs; however, they only examined isolated anatomical trunk motions in the plane of motion,^21^ found inaccurate trunk orientation estimates relative to the pelvis during sports motions,^22^ examined a sports motion with limited trunk range of motion,^23^ or presented limited data and assessed only specific phases of the motion with limited duration.^24^ Thus, the accuracy of three‐dimensional trunk orientation relative to the pelvis measured using lightweight and wireless IMUs during dynamic sports motions and clinical tasks, such as isolated anatomical trunk motion, remains to be established.

The first objective of this study was to evaluate the accuracy of the three‐dimensional trunk orientation relative to the pelvis measured using lightweight and wireless IMUs during dynamic sports motions. The second objective was to evaluate the accuracy of the IMU‐based three‐dimensional trunk orientation relative to the pelvis measured during isolated anatomical trunk motion, as a potential clinical tool to assess trunk range of motion.

## 2 METHODS

### 2.1 Procedure

The experimental procedure was approved by the University of Auckland Human Participants Ethics Committee (UAHPEC). Ten healthy male participants volunteered for this study (mean age: 28.50 ± 6.36 years; height: 1.77 ± 0.10 m; body mass 72.11 ± 5.33 kg). Before data collection, written informed consent was provided by all participants and the IMUs and motion capture markers were attached. First, a static trial with the anatomical neutral pose was recorded. Subsequently, participants were asked to perform four sports motions as powerful as they could: a golf swing; a one‐handed ball throw; a tennis serve; and a baseball swing. Additionally, isolated anatomical trunk motions were recorded separately: flexion/extension, left/right lateral flexion, or left/right axial rotation. Each trial contained one (eg, flexion, extension) or two (eg, flexion, extension, flexion, extension) cycles of motion over the maximal voluntary range of motion. In order to limit out of plane motion, before each anatomical trunk motion trial, the participants were instructed to move their arms as follows: in line with the ventral side of their lower limbs (flexion/extension); in line with the lateral side of their trunk and lower limbs (lateral flexion); or horizontally in T‐pose (axial rotation). The participants were instructed to remain static until after the recording was started. Each trial was recorded twice. If the performed motion was not in agreement with the instructions or when there was no Bluetooth connection at the start or end of the trial, the trial was excluded and a third recording of the motion was performed. After data collection, successful recordings were selected for analysis.

### 2.2 Equipment

Twelve optoelectronic Vicon V16 cameras operating at 100 Hz (VICON, Oxford, UK) were used to track retro‐reflective motion capture markers. Two IMUs (IMU BlueThunder, IMeasureU, Auckland, New Zealand) were used. Each IMU (dimension: 40 × 28 × 15 mm; weight: 12 g) included a three‐axis accelerometer (range ±16 G), three‐axis gyroscope (range ±2000°/s), and three‐axis magnetometer (range ±1200 μT). The raw IMU data were stored on‐board at 100 Hz and synchronized with the marker data via a Bluetooth connection using build‐in synchronization of the Nexus motion capture software (version 2.7). After data collection, the IMU data were uploaded from the IMU to the Nexus motion capture software. Gaps in the marker trajectories were interpolated using the Nexus motion capture software. Kinematic analysis was performed using custom code written in MATLAB (2016b, The MathWorks, Inc., US).

### 2.3 Marker placement

Eight retro‐reflective motion capture markers were placed on the following anatomical landmarks; right and left anterior superior iliac spines (RASIS and LASIS, respectively), right and left posterior superior iliac spines (RPSIS and LPSIS, respectively), xiphoid process (XP), suprasternal notch (SN), spinous process of the 7th cervical vertebra (C7), and spinous process of the 8th thoracic vertebra (T8). Three retro‐reflective motion capture markers were attached to each IMU (see Appendix 2).

### 2.4 IMU placement

One IMU was placed on the pelvis; the midpoint between RPSIS and LPSIS, and a second IMU was fixed to the trunk; the spinous process of the first thoracic vertebra (T1). The IMUs were mounted on a 3D printed clip with a flat base which was attached to the skin using double‐sided adhesive tape. The present study aimed to measure the three‐dimensional orientation of the trunk relative to the pelvis. Since the trunk is a non‐rigid segment, the most proximal spinal bony landmark of the trunk (ie, T1) was selected, in contrast to sensor placement recommendations for measuring the trunk inclination.^25^

### 2.5 Data analysis

#### 2.5.1 MOCAP‐based trunk orientation

The motion capture (MOCAP) data analysis consisted of two parts. First, the anatomical planes of the thorax, pelvis, and trunk were defined. Second, the marker‐based trunk orientation was calculated using the markers attached to the IMUs, resulting in what was considered to be the “gold standard” MOCAP‐based trunk orientation.

##### Trunk anatomical coordinate system

**FIGURE 1:**
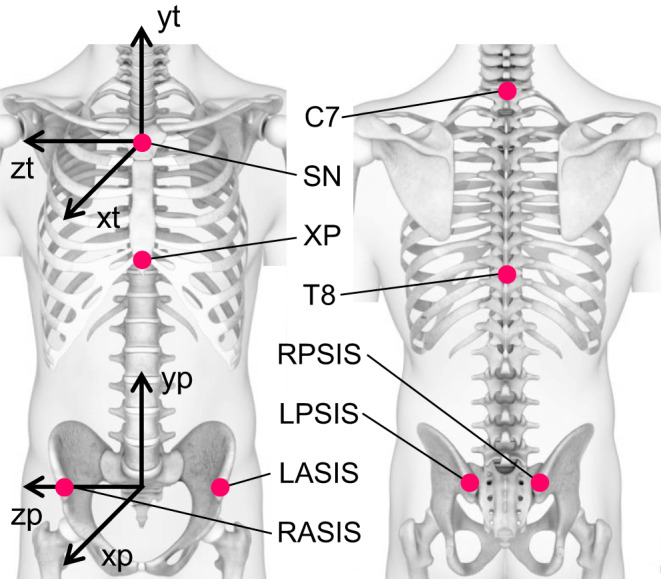
Anatomical coordinate systems (ACSs) of the thorax (xt, yt, and zt pointing ventral, proximal, and laterally to the right, respectively) and pelvis (xp, yp, and zp pointing ventral, proximal, and laterally to the right, respectively) and the corresponding anatomical landmarks (SN: suprasternal notch; XP: xiphoid process; C7: spinous process of the 7th cervical vertebra; T8: spinous process of the 8th thoracic vertebra; RASIS and LASIS: right and left anterior superior iliac spines, respectively; and RPSIS and LPSIS: right and left posterior superior iliac spines, respectively)

Using the three‐dimensional locations of the markers placed at anatomical landmarks, the anatomical coordinate systems (ACSs) of pelvis and thorax were constructed. The pelvis ACS was calculated using the markers placed on RPSIS, LPSIS, RASIS, and LASIS,^26^ and the thorax ACS was constructed with the markers placed on XP, SN, C7, and T8 (Figure 1).^27^ The joint coordinate system of the trunk was defined as the thorax ACS relative to the pelvis ACS.^28^

##### Technical coordinate system‐based trunk orientation

The three‐dimensional locations of the markers attached to the IMUs were used to construct technical coordinate systems (TCSs) fixed to the T1 IMU and pelvis IMU (T1 TCS and pelvis TCS, respectively).^29^ Subsequently, the T1 TCS and the pelvis TCS were aligned with the thorax and pelvis ACS. The TCS‐based trunk orientation with respect to the global marker coordinate system was represented by the orientation of the aligned T1 TCS relative to the aligned pelvis TCS. The final MOCAP‐based trunk orientation with respect to the anatomical neutral pose was obtained by correcting each trial’s TCS‐based trunk orientation with respect to the global marker coordinate system for the offset of the TCS‐based trunk orientation with respect to the global marker coordinate system during the anatomical neutral pose trial.

#### 2.5.2 IMU data analysis

##### Data fusion algorithm

###### Algorithm

The orientation of each IMU relative to the global earth coordinate system was computed by combining the raw accelerometer, gyroscope, and magnetometer data from each IMU using a data fusion algorithm.^14^ The computation of the IMU orientation relies on the fusion of two orientation estimates: the numerical integration of the angular velocity and the orientation relative to the gravity vector and the direction of the earth magnetic field. The algorithm computes an optimized fusion of the orientation estimates to minimize the effect of these errors on the accuracy of the computed IMU orientation. The fusion can be controlled with the algorithm’s input parameter *β*. The value of *β* (0 to 1) determines the tradeoff between the algorithm’s two orientation estimates. An increased *β* results in a more accelerometer‐ and magnetometer‐based IMU orientation computation, and a decreased *β* leads to a more gyroscope‐based IMU orientation computation.

###### Initial guess

The described data fusion algorithm commences, starting from an initial guess, with the convergence of the IMU orientation to its orientation in the global earth coordinate system (orientation convergence). For the duration of this phase, the computed IMU orientation is likely inaccurate, so ideally one should start each recording with a period of static pose. For each trial, an initial guess of each IMU orientation was computed using the data fusion algorithm to minimize the duration of the orientation convergence. Starting with the IMU orientation during the anatomical neutral pose, the initial guess of the IMU orientation was estimated based on the accelerometer and magnetometer data of the trial’s first sample and zero three‐dimensional angular velocity. We assumed that during the first sample of each trial, the participants retained a static pose, so that both IMUs were in static orientation. For the estimation of the initial guess, the *β* of the data fusion algorithm was set to 0.9 (ie, the orientation computation is highly dependent on the accelerometer and magnetometer measurement). An additional effect of a *β* of 0.9 was a high convergence rate (orientation convergence rate increases with *β*).

##### IMU‐based trunk orientation

For each trial, the orientations of the T1 IMU and pelvis IMU relative to the global earth coordinate system expressed as quaternion (${}^{G}q_{t1}$ and ${}^{G}q_{p}$, respectively) were estimated with the data fusion algorithm. Moreover, the quaternion orientation of each IMU relative to the global earth coordinate system during the anatomical neutral pose was estimated (${}^{G}q_{t1,static}$ and $\;{}^{G}q_{p,static}$). Equation (1) describes the calculation of the pelvis IMU orientation with respect to the anatomical neutral pose (${}^{G}q_{p/static}$). The ⊗ marks the Hamilton quaternion product, a quaternion written with the superscript −1 (eg, *q* ^−1^) signifies the quaternion inverse.

(1) $${}^{G}q_{p/static}\;=\;{}^{G}q_{p/static}^{‐1}\;⊗\;{}^{G}q_{p}$$

To align the T1 IMU with the pelvis IMU, the orientation of the T1 IMU relative to the pelvis IMU during the anatomical neutral pose (${}^{G}q_{t1/p,static}$) was determined from the T1 and pelvis accelerometer data; the rotation to align the T1 with the pelvis gravity vector was evaluated under the assumption participants remained static during the anatomical neutral pose trial; thus, the accelerometers only measured gravity. Equation (2) describes the calculation of the T1 IMU orientation with respect to the anatomical pose and subsequently the rotation to align the T1 IMU with the pelvis IMU.’

(2) $${}^{G}q_{t1/static}\;=\;{}^{G}q_{t1/p,static}^{‐1}\;⊗\;\left({}^{G}q_{t1,static}^{‐1}\;⊗\;{}^{G}q_{t1}\right)$$

Equations (3) and (4) describe the alignment of the calculated IMU orientations, ${}^{G}q_{p/static}$ and ${}^{G}q_{t1/static}$ (equation (1) and (2)), with the pelvis and thorax ACS, respectively, using the predefined relationships between the IMU coordinate system and the relevant TCS constructed with the markers attached to the IMU (see Appendix 1). The rotations to align the orientation of the pelvis IMU with the pelvis ACS and the T1 IMU with the thorax ACS are represented by ${}^{G}q_{plMU/pACS}$ and ${}^{G}q_{t1lMU/thACS}$, respectively.

(3) $${}^{G}q_{p/pACS}\;=\;{}^{G}q_{p/static}\;⊗{}^{G}q_{pIMU/pACS}$$

(4) $${}^{G}q_{t1/thACS}\;={}^{G}q_{t1/static}⊗{}^{G}q_{t1IMU/thACS}$$

The IMU‐based trunk orientation aligned with the trunk ACS was represented by the orientation of ${}^{G}q_{t1/thACS}$ relative to ${}^{G}q_{p/pACS}$, as described by Equation (5).

(5) $${}^{G}q_{trunk/trACS}\;=\;\;Gq_{p/ACS}^{‐1}\;⊗\;{}^{G}q_{t1/thACS}$$

For the IMU‐based trunk orientation, the orientation offset between the T1 and pelvis IMU during the anatomical neutral pose was considered as anatomically “zero.” Accordingly, the orientation offset between the T1 and pelvis IMU during the anatomical neutral pose was aligned with the trunk ACS and subsequently multiplied with ${}^{G}q_{trunk/trACS}$, as described by Equation (6).

(6) $${}^{G}q_{\mathrm{trunk}}\;={\left(\;Gq_{pIMU/pACS}^{‐1}\;⊗\;({}^{G}q_{t1/p,static}^{‐1}\;⊗{}^{G}q_{t1IMU/thACS}\right)}^{‐1}\;⊗{}^{G}q_{trunk/trACS}$$

Eventually, the quaternion ${}^{G}q_{\mathrm{trunk}}$ represents the IMU‐based trunk orientation aligned with the trunk ACS with respect to the anatomical neutral pose.

#### 2.5.3 Cardan angles and filter characteristics

For each trial, the time series of the MOCAP‐ and IMU‐based trunk quaternion orientation were transformed into time series of rotation matrices. Subsequently, the rotation matrices were decomposed into three Cardan angles using the ZXY decomposition format. According to the recommended definition of the anatomical planes of the trunk, the X, Y, and Z angles represent lateral flexion, axial rotation, and flexion/extension, respectively.^27^ In contrast to previous recommendations regarding the assessment of the three‐dimensional accuracy of inertial sensors,^30^ the present study computed the Cardan angles considering the practical application of the current method to measure the three‐dimensional trunk orientation relative to the pelvis. The trunk angles of the dynamic sports motions and anatomical trunk motions were filtered with a second order zero‐lag low‐pass Butterworth filter with a cutoff frequency of 6 Hz. The cutoff frequency was selected based on the assumption that frequencies higher than 6 Hz in kinematic data were representing noise rather than human movement.^31^, ^32^

#### 2.5.4 Tuning the data fusion algorithm

The value of the data fusion algorithm’s input parameter β was determined in an analysis on the anatomical trunk motion trials (see Appendix 3). A *β* of 0.034 was used as input for the data fusion algorithm in this study. This value was similar to the β presented by Madgwick and colleagues.^14^

#### 2.5.5 Measures of accuracy and statistical comparison

For every trial, the RMSE was computed between the IMU‐ and MOCAP‐based flexion/extension, lateral flexion, and axial rotation angles. The RMSE results were interpreted using previously proposed clinical guidelines: RMSEs of 2° or less indicate an acceptable accuracy, RMSEs between 2° and 5° signify a reasonable accuracy, and RMSEs of 5° or higher suggest an unacceptable accuracy and consequently require careful interpretation.^33^ Additionally, for every trial, to evaluate the similarity between the IMU‐ and MOCAP‐based trunk flexion/extension, lateral flexion, and axial rotation angles, the Pearson correlation coefficient (R) and the coefficient of multiple determination (*R* ^2^; the square of *R*) were determined. Previously published guidelines regarding the interpretation of *R* values were used: 0.65‐0.75 moderate; 0.75‐0.85 good; 0.85‐0.95 very good; 0.95‐1 excellent.^34^

The mean flexion/extension, lateral flexion, and axial rotation RMSE, *R* and *R* ^2^ with standard deviation, and 95% confidence interval were computed across all trials and separately for the dynamic sports motion, anatomical trunk motions, flexion/extension, lateral flexion, and axial rotation trials.

## 3 RESULTS

**FIGURE 2:**
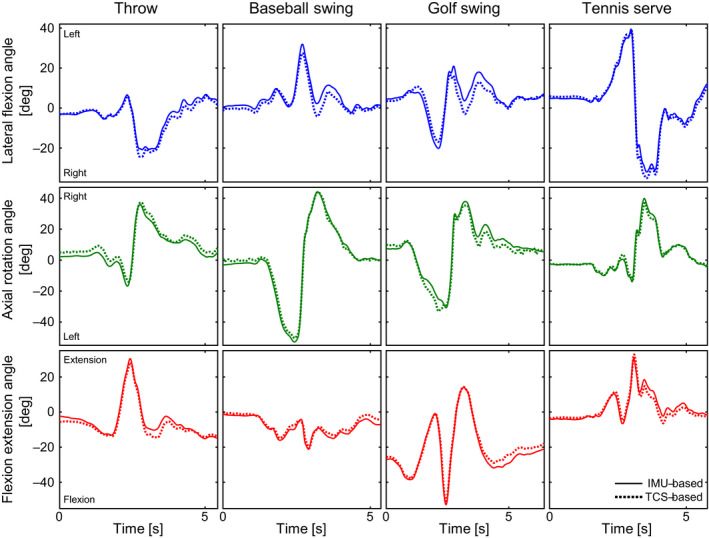
The IMU‐based (solid) and MOCAP‐based (dotted) trunk angles over time. The columns represent the four dynamic sports motions: throw (participant 1); baseball swing (participant 10); golf swing (participant 9); tennis serve (participant 4). The rows represent the three trunk angles in degrees: lateral flexion (blue; positive and negative are left and right, respectively); axial rotation (green; positive and negative are right and left, respectively); flexion extension (red; positive and negative are extension and flexion, respectively)

In total, 71 sports motions (19 golf swings, 15 one‐handed ball throws, 19 tennis serves, and 18 baseball swings; a minimum of one trial per participant) were recorded. The analysis across all dynamic sports motion trials yielded a mean RMSE of 3.7 ± 1.5, 4.9 ± 3.1, and 3.0 ± 1.3 degrees (Table 1); a mean *R* of 0.90 ± 0.12, 0.99 ± 0.01, and 0.97 ± 0.05 (Table 2); and a mean *R* ^2^ of 0.82 ± 0.19, 0.98 ± 0.02, and 0.95 ± 0.09 (Table 3) for lateral flexion, axial rotation, and flexion/extension angles, respectively. Figure 2 illustrates the accuracy of the trunk angles determined using IMUs compared to the MOCAP‐based trunk angles during the throw (first column), baseball swing (second column), golf swing (third column), and tennis serve (fourth column).

**TABLE 1:** Mean RMSE between the IMU‐ and MOCAP‐based lateral flexion, axial rotation, and flexion/extension angles for the dynamic sports motion, anatomical trunk motion, lateral flexion, axial rotation, and flexion/extension trials and for all trials (n = 196). Standard deviation (S.D.) and 95% confidence interval (95% CI) are also reported.

Trial type	RMSE for each trunk angle [deg]								
	Lateral flexion			Axial rotation			Flexion/extension		
	Mean	S.D.	95% CI	Mean	S.D.	95% CI	Mean	S.D.	95% CI
Dynamic sports motions	3.7	1.5	3.4‐4.0	4.9	3.1	4.1‐5.6	3.0	1.3	2.7‐3.3
Anatomical trunk motions	2.6	1.0	2.3‐2.8	4.5	2.0	4.0‐4.9	3.0	1.5	2.8‐3.3
Lateral flexion	2.5	0.7	2.1‐2.9	5.3	2.5	4.5‐6.0	4.5	1.4	4.0‐4.9
Axial rotation	2.5	0.7	2.1‐2.9	4.2	1.7	3.4‐5.0	1.9	0.6	1.5‐2.4
Flexion/extension	2.7	1.4	2.3‐3.1	3.9	1.3	3.1‐4.7	2.6	0.9	2.2‐3.1
All trials	3.0	1.3	2.8‐3.2	4.6	2.4	4.3‐4.9	3.0	1.4	2.8‐3.2

**TABLE 2:** Mean R (Pearson’s correlation coefficient) and standard deviation (S.D.) between the IMU‐ and MOCAP‐based lateral flexion, axial rotation, and flexion/extension angles for the dynamic sports motion, anatomical trunk motion, lateral flexion, axial rotation, and flexion/extension trials and for all trials (n = 196).

Trial type	R for each trunk angle					
	Lateral flexion		Axial rotation		Flexion/extension	
	Mean	S.D.	Mean	S.D.	Mean	S.D.
Dynamic sports motions	0.90	0.12	0.99	0.01	0.97	0.05
Anatomical trunk motions	0.90	0.17	0.77	0.31	0.95	0.06
Lateral flexion	1.00	0.00	0.81	0.22	0.91	0.08
Axial rotation	0.96	0.05	0.99	0.01	0.96	0.03
Flexion/extension	0.74	0.21	0.50	0.35	1.00	0.00
All trials	0.90	0.15	0.85	0.27	0.96	0.06

**TABLE 3:** Mean *R* ^2^ (coefficient of multiple determination) and standard deviation (S.D.) between the IMU‐ and MOCAP‐based lateral flexion, axial rotation, and flexion/extension angles for the dynamic sports motion, anatomical trunk motion, lateral flexion, axial rotation, and flexion/extension trials and for all trials (n = 196).

Trial type	R^2^ for each trunk angle					
	Lateral flexion		Axial rotation		Flexion/extension	
	Mean	S.D.	Mean	S.D.	Mean	S.D.
Dynamic sports motions	0.82	0.19	0.98	0.02	0.95	0.09
Anatomical trunk motions	0.84	0.24	0.69	0.35	0.91	0.11
Lateral flexion	1.00	0.00	0.71	0.28	0.83	0.13
Axial rotation	0.93	0.08	0.99	0.01	0.92	0.05
Flexion/extension	0.60	0.28	0.37	0.32	1.00	0.00
All trials	0.83	0.22	0.80	0.31	0.93	0.10

**FIGURE 3:**
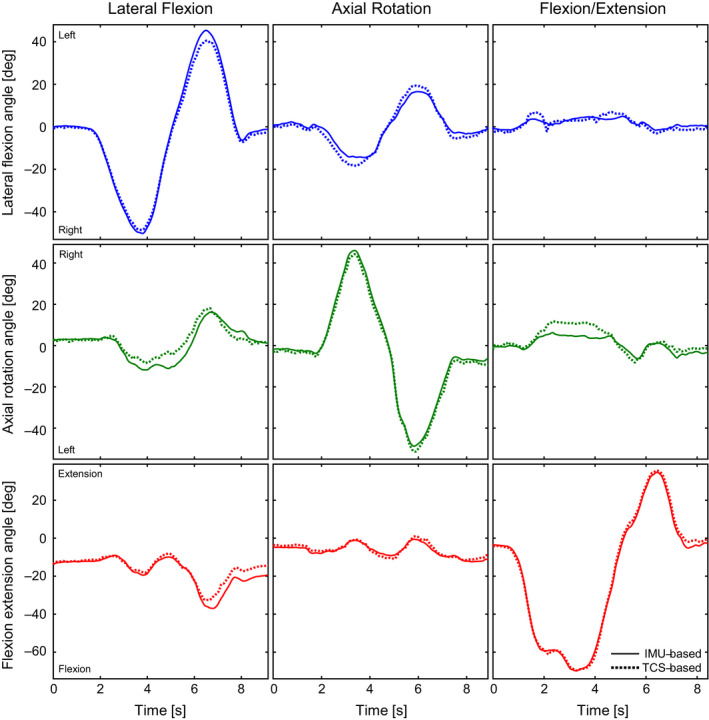
The IMU‐based (solid) and MOCAP‐based (dotted) trunk angles over time. The columns represent three anatomical trunk motion trials: lateral flexion (participant 7); axial rotation (participant 10); flexion/extension (participant 2). The rows represent the three trunk angles in degrees: lateral flexion (blue; positive and negative are left and right, respectively); axial rotation (green; positive and negative are right and left, respectively); flexion extension (red; positive and negative are extension and flexion, respectively)

A total of 125 anatomical trunk motions (42, 41, and 42 trials of lateral flexion, axial rotation, and flexion/extension, respectively) were recorded. The analysis across all anatomical trunk motion trials yielded a mean RMSE of 2.6 ± 1.0, 4.5 ± 2.0, and 3.0 ± 1.5 degrees (Table 1); a mean R of 0.90 ± 0.17, 0.77 ± 0.31, and 0.95 ± 0.06 (Table 2); and a mean R^2^ of 0.84 ± 0.24, 0.69 ± 0.35, and 0.91 ± 0.11 (Table 3) for lateral flexion, axial rotation, and flexion/extension angles, respectively. The mean RMSE, *R*, and *R* ^2^ across each anatomical trunk motion separately are also presented in Tables 1, 2, 3, respectively. Figure 3 illustrates the accuracy of the trunk angles determined using IMUs compared to the MOCAP‐based trunk angles during the anatomical trunk motion trials: lateral flexion (first column); axial rotation (second column); and flexion/extension (third column).

Lastly, the analysis across all trials yielded a mean RMSE of 3.0 ± 1.3, 4.6 ± 2.4, and 3.0 ± 1.4 degrees (Table 1); a mean *R* of 0.90 ± 0.15, 0.85 ± 0.27, and 0.96 ± 0.06 (Table 2); and a mean *R* ^2^ of 0.83 ± 0.22, 0.79 ± 0.31, and 0.93 ± 0.10 (Table 3) for lateral flexion, axial rotation, and flexion/extension angles, respectively.

## 4 DISCUSSION

The aim of this study was to assess the accuracy of the three‐dimensional trunk orientation relative to the pelvis determined using lightweight and wireless IMUs during dynamic sports motions. The RMSE values between the IMU‐ and MOCAP‐based trunk angles were on average less than 5 degrees around all three axes (Table 1). Considering the described RMSE guidelines,^33^ these RMSE values indicate a reasonable accuracy. On average, the lateral flexion angles showed similarity interpreted as “very good” and the similarity of both the axial rotation and flexion/extension angles should be interpreted as “excellent” according to the described guidelines regarding the value of *R*.^34^ The *R* ^2^ results indicate that during the dynamic sports motions on average 82%, 98%, and 95% of the variance of the MOCAP‐based lateral flexion, axial rotation, and flexion/extension angle, respectively, can be explained by the trunk angles estimated using lightweight and wireless IMUs (Table 3). Compared to the present study, in alpine skiing, better accuracy and impaired similarity was found previously for the three‐dimensional trunk orientation relative to the pelvis measured using wireless IMUS. However, the range of trunk motion during the alpine skiing trials was limited. Additionally, this may have resulted in a low signal‐to‐noise ratio and thus explain the impaired similarity results.^23^ In cricket bowling, no significant differences were found between MOCAP‐ and IMU‐based three‐dimensional trunk orientation.^24^ However, no accuracy and similarity measures were presented and the trunk angles were compared only during specific phases of the movement, thus for a limited duration. In discus throwing, impaired results were found, potentially due to the use of the MOCAP‐based orientation of the trunk anatomical coordinate system as “gold standard” reference whereas the present study selected the technical coordinate system‐based trunk orientation.^22^

Additionally, the accuracy of the three‐dimensional trunk orientation relative to the pelvis determined with lightweight and wireless IMUs during a clinical task was verified in trials of isolated anatomical trunk motion. On average, both overall and for each anatomical trunk motion separately, the IMU‐based trunk angles showed a reasonable accuracy with RMSE values of less than 5 degrees (except the axial rotation angle during lateral flexion). Similar RMSE values have been reported in the literature for box lifting and the timed‐up‐and‐go walking assessment using a system of heavy, wired IMUs and a logging device to measure the three‐dimensional trunk orientation relative to the pelvis.^16^, ^20^ Previous studies presented RMSE values lower than 3 degrees^15^, ^18^, ^19^, ^21^ or higher than 5 degrees^17^ indicating a better or impaired accuracy when compared to results in the present study. These comparisons are limited, however, since several studies considered the segmental orientation (ie, orientation relative to a global coordinate system) of the thorax.^17^, ^18^, ^19^ So, the accuracy of only one IMU attached to the trunk is assessed whereas the present study assessed the accuracy of the IMU‐based trunk orientation based on the cumulative effect of the accuracy of two IMUs. Recently, RMSE values on average lower than 3 degrees were presented for the trunk orientation relative to the pelvis measured using wireless IMUs.^21^ However, in contrast to the present study, the accuracy and similarity measures of each anatomical trunk motion were only provided for the plane of motion.

During the anatomical trunk motions, the IMU‐based trunk angles showed generally “very good” to “excellent” similarity (*R* > 0.90), especially in the plane of motion (R ≥ 0.99). Similarly, *R* ^2^ values of 0.99 or higher are found in the plane of motion, which indicated that 99% or more of the variance of the MOCAP‐based trunk angles can be explained by the trunk angles estimated using the current IMUs. The presented *R* values coincide with previous findings during box lifting, the timed‐up‐and‐go walking assessment, and isolated anatomical trunk motions.^15^, ^16^, ^20^, ^21^ Decreased mean *R* and *R* ^2^ values were found out of the plane of motion (lateral flexion and axial rotation angle during flexion/extension and axial rotation angle during lateral flexion). This could be related to a decreased signal‐to‐noise ratio in the IMU‐ and MOCAP‐based trunk angles out of the plane range of motion,^35^ especially during flexion/extension (Figure 3; see Appendix 4). Namely, a decreased range of motion, which in particular would be expected out of the plane of motion during isolated anatomical trunk motions, could result in decreased *R* and *R* ^2^ values.^20^

In some isolated anatomical trunk motion trials, considerable motion out of the plane of motion was observed. This could be attributed to skin movement artifacts, especially at T1, due to movement of the underlying vertebra and muscles. Additionally, the trunk is comprised of multiple vertebrae and intervertebral joints introducing multiple degrees of freedom within the trunk segment which may complicate performing isolated anatomical trunk motions only in the intended plane of motion.

On average over all trials, “very good” to “excellent” similarity was observed between the IMU‐ and MOCAP‐based trunk angles (*R* ≥ 0.85). In line with previously discussed findings, an overall mean RMSE of less than 5 degrees was found for the three trunk angles. The overall accuracy of the three‐dimensional trunk angles estimated using lightweight and wireless IMUs is considered reasonable.

In contrast to the IMU systems previously presented in the literature,^15^, ^16^, ^17^, ^18^, ^19^, ^20^ the current study used lightweight and wireless IMUs to measure the three‐dimensional trunk orientation relative to the pelvis during dynamic sports motions and the anatomical trunk motions. This provides the opportunity to perform future measurements of the three‐dimensional trunk orientation outside the laboratory. However, the acceptable limit of accuracy of the IMU‐based trunk orientation measurement could depend on the measurement aim. So, for every context, one should determine whether the current IMUs meet the accuracy demands to measure the three‐dimensional trunk orientation.

Estimating the initial guess of the IMU orientation, based on the assumption that participants retained a static pose during the first sample of each trial, can be considered a limitation of the current study. The current *β* value of 0.034 involved a prolonged orientation convergence of the data fusion algorithm, and an error in the initial guess could therefore remain as an error in the IMU orientation over time. So, the accuracy of the algorithm’s IMU orientation estimate relied considerably on the level of accuracy of the initial guess. The accuracy of the initial guess could be reduced as a result of movement of the participant at the start of the trial. Any resulting linear accelerations may have affected the algorithm’s computation of the orientation with respect to the gravity vector since at the start of each trial the initial guess estimation relied primarily on the accelerometer and magnetometer data. This would be reflected predominantly in the axial rotation angle since the IMU‐based trunk Y axis (aligned with the trunk ACS Y axis) pointed approximately vertically at the start of each trial. In several dynamic sports motion and anatomical trunk motion trials, motion of the trunk of the participants was observed at the start of the measurement which may have caused an inaccurate initial guess and consequently an increased RMSE between the IMU‐ and MOCAP‐based trunk orientation. Despite this limitation, the present study revealed that the three‐dimensional IMU‐based trunk orientation can be measured with a reasonable accuracy. During future measurements, at least three seconds of a static pose should be recorded at the start of each measurement to enable a longer time span over which the initial guess is estimated. Consequently, improved accuracy of the initial guess could be established and potentially even higher levels of accuracy of the IMU‐based trunk orientation measurement could be produced.

Tuning of the data fusion algorithm is essential since the value of β affects the level of accuracy of the IMU‐based trunk orientation. The present study used the MOCAP‐based trunk orientation as a “gold standard” measure to obtain the optimal β value for measuring the trunk orientation with a reasonable accuracy using the current IMUs. Future studies could investigate methods to tune the data fusion algorithm without the need of a MOCAP reference measurement to improve the practicality of using IMUs to measure the trunk orientation.

The present study used a single *β* value optimized for the combined use of two IMUs and realized reasonable accurate estimations of the IMU‐based trunk orientation which showed very good to excellent similarity compared with the MOCAP‐based trunk orientation. Since the gyroscope measurement error is different per IMU, ideally, an individualized optimal β value should be determined for every IMU. This could potentially result in even higher levels of accuracy of the trunk orientation estimates.

Although the dynamic sports motions examined in the present study involved considerable trunk range of motion, these motions contained limited translation and consequently linear acceleration. The gravity vector may become indistinguishable from linear accelerations, and hence, the performance of the fusion algorithm may decrease. Future studies should evaluate the accuracy of three‐dimensional trunk orientation measured using the current IMUs during dynamic sports motions with considerable linear accelerations.

Future research should focus on introducing an accurate method to align the IMUs with the anatomical planes of the trunk (ie, anatomical calibration). The anatomical trunk angles lateral flexion, axial rotation, and flexion/extension can then be correctly calculated. As a result, kinematic cross‐talk could be minimized and the repeatability of the trunk orientation measurements could be assured.^23^, ^36^, ^37^ Such an alignment method would improve the practicality of measuring the three‐dimensional trunk orientation relative to the pelvis using the current IMUs.

## 5 PERSPECTIVE

The three‐dimensional trunk orientation relative to the pelvis measured using wireless and lightweight IMUs during dynamic sports motions and the anatomical trunk motions showed a reasonable accuracy and very good to excellent similarity compared to the motion capture‐based trunk orientation. When aligned to the relevant segment, the current IMUs are a promising alternative to the motion capture system and previously presented IMU‐based systems, in particular for the field‐based, measurement of the three‐dimensional trunk orientation during dynamic sports motions and the anatomical trunk motions.

These measurements could be used for improving sports performance, prevention or rehabilitation of sports related injuries, or clinically relevant questions. For in‐field use of the current IMUs, sensor‐to‐segment calibration is required and a three second period of static pose at the start of every trial is recommended to enable the fusion algorithm to compute accurate IMU orientation estimates. Although the present study compared the accuracy and similarity results to established (clinical) guidelines and previous research, the accuracy demands of the measurement device may differ with the context. For example, these demands could be based on the expected range of motion or the sensitivity of a biomechanical model to orientation errors. Eventually, it is up to the researcher whether the current IMUs meet the accuracy demands.

## Conflict of interest

Thor Besier is a consultant to IMeasureU.

## Figures and Tables

**FIGURE S1:**
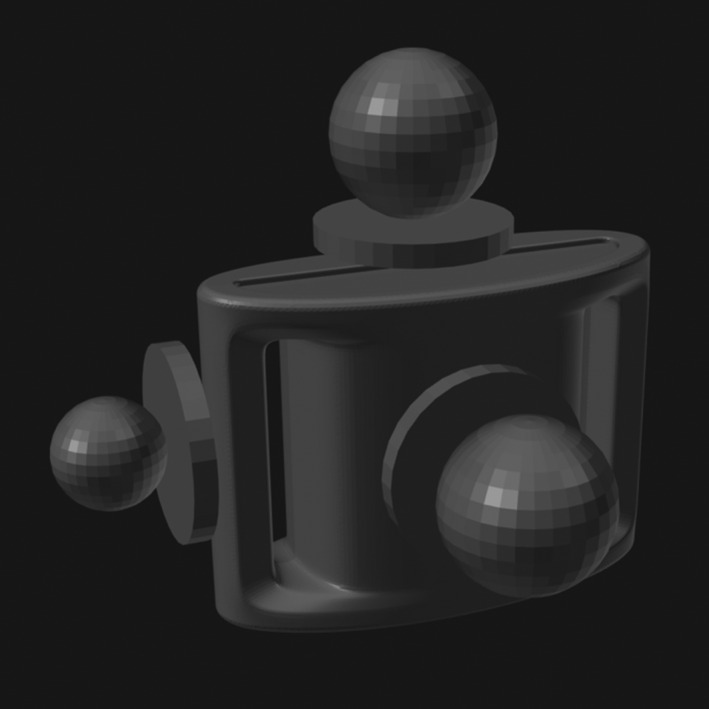
Three‐dimensional model (scale 1:1) of an IMU with three motion capture markers (two 14 mm markers, one 9.5 mm marker) displayed in Blender (Blender Foundation, Amsterdam, Netherlands). The 3D printed clip, which was located between the IMU and the skin of the participants, is not displayed

**FIGURE S2:**
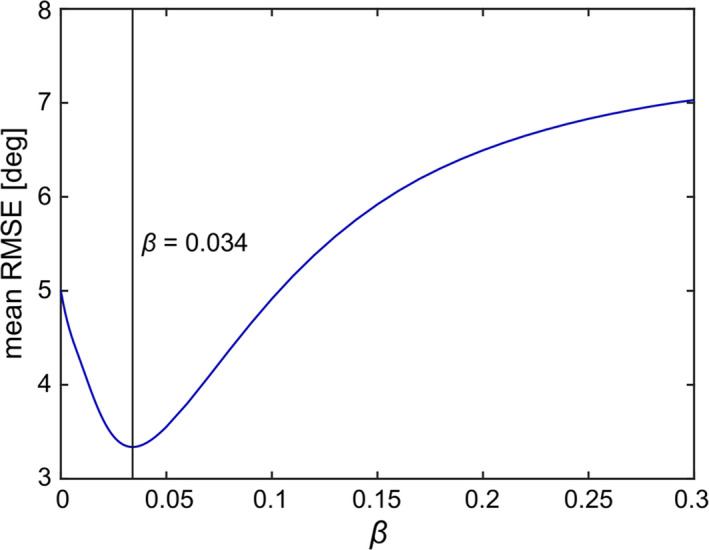
Data fusion algorithm performance. The mean RMSE between the IMU‐ and MOCAP‐based trunk angles across the three trunk angles across all included anatomical trunk motion trials is displayed on the vertical axis. The *β* value used as input for the data fusion algorithm to compute the IMU‐based trunk orientation is displayed at the horizontal axis. The vertical line represents the *β* value (*β* = 0.034) at the mean RMSE minimum (mean RMSE = 3.49 ± 1.14º)

## APPENDIX 1 Orientation of the IMU in the global marker coordinate system

*Local marker coordinates*. The three‐dimensional coordinates of the center of each motion capture marker in the relevant IMU coordinate system were estimated based on a three‐dimensional model of an IMU with three motion capture markers (scale 1:1; Figure Appendix 2) created in Blender (Blender Foundation, Amsterdam, Netherlands). These model‐based local coordinates of the motion capture markers were used to create a model of each IMU with three motion capture markers (scale 1:1) in OpenSim (Simbios, Stanford, US). With an analysis in OpenSim (Adjust Model Markers; Scale Tool), the location of each motion capture marker relative the relevant IMU coordinate system was adjusted to match the location recorded during a static trial (two IMUs placed on the ground, approximately in the center of the measurement volume of the optoelectronic camera system). During the analysis, the error between the locations of the model markers (ie, estimated using Blender) and the real world markers (ie, static trial) was minimized. The adjusted locations of the motion capture markers in the relevant IMU coordinate system were assumed constant for all measurements. For each participant, these marker locations were used to align the pelvis and T1 IMU with the pelvis and thorax ACS, respectively.

*Alignment*. The TCSs of the T1 and the pelvis IMU in the relevant IMU coordinate system were calculated with the adjusted three‐dimensional local marker locations. The orientation of each TCS in the relevant IMU coordinate system was assumed to be constant during all measurements. Hence, the rotation matrices were calculated to align the T1 and the pelvis IMU local marker coordinate systems with the global marker coordinate systems of the T1 and the pelvis IMU, respectively. Subsequently, based on the anatomical neutral pose, the rotations were calculated to align the orientations of the IMUs with the ACSs. Equation [7] describes the calculation of the rotation matrix to align the IMU with the relevant ACS.

(7) $${}^{\mathrm{ACS}}R_{\mathrm{IMU}}\;=\;{\left({}^{\mathrm{glob}}R_{\mathrm{TCS}}\cdot {}^{\mathrm{loc}}R_{\mathrm{TCS}}^{T}\right)}^{T}\cdot {}^{\mathrm{glob}}R_{\mathrm{ACS}}$$

With ${}^{\mathrm{glob}}R_{\mathrm{TCS}}$ as the orientation of the relevant TCS in the global marker coordinate system, $\;locR_{\mathrm{TCS}}^{T}$ as the transpose of the orientation of the relevant TCS in the IMU coordinate system, ${}^{\mathrm{glob}}R_{\mathrm{ACS}}$ as the orientation of the relevant ACS in the global marker coordinate system, and ${}^{\mathrm{ACS}}R_{\mathrm{IMU}}$ as the rotation matrix to align the IMU with the relevant ACS.

## APPENDIX 3 Tuning the data fusion algorithm

The value of the data fusion algorithm’s input parameter β was determined in an analysis on the anatomical trunk motion trials. The IMU‐based trunk angles of the anatomical trunk motions were calculated using a range of *β* values (*β* = [10^−6^ to 0.3]). The root‐mean‐square error (RMSE) between the IMU‐ and MOCAP‐based trunk angles was considered a measure of accuracy with a low RMSE indicating a high level of accuracy. The optimal *β* value with respect to the combined gyroscope measurement error of the two current IMUs was defined as the *β* belonging to the minimum mean RMSE across the three trunk angles, across all included trials.

Twenty trials were randomly selected from every anatomical trunk motion trial subgroup: one or two cycles of flexion/extension, lateral flexion, or axial rotation over the maximal voluntary range of motion. We assumed that the potential effect of the difference in gyroscope measurement error along the different anatomical axes was therefore equally expressed in the minimum mean RMSE across all included trials. Hence, a total of 120 anatomical trunk motion trials were included in the analysis.

According to the analysis, a *β* of 0.034 yielded the minimum mean RMSE of 3.3 ± 1.1º across all included anatomical trunk motion trials (see Figure Appendix 3). This value was therefore used as input for the data fusion algorithm in this study. This value was similar to the *β* presented by Madgwick and colleagues.^14^

## APPENDIX 4

**TABLE S1** Mean and standard deviation (S.D.) of the average MOCAP‐based lateral flexion, axial rotation, and flexion/extension angles for the dynamic sports motion, anatomical trunk motion, lateral flexion, axial rotation, and flexion/extension trials and for all trials (n = 196)

**Table:**

Trial type	Average MOCAP‐based trunk angle [deg]					
	Lateral Flexion	Axial Rotation	Flexion/extension			
	Mean	S.D.	Mean	S.D.	Mean	S.D.
Dynamic sports motions	8.0	3.0	15.2	5.5	17.5	8.3
Anatomical trunk motions	10.6	8.7	10.1	7.9	19.3	13.3
Lateral flexion	22.0	4.0	6.6	2.4	14.3	5.0
Axial rotation	6.5	1.8	20.1	4.6	7.7	2.7
Flexion/extension	3.1	1.1	3.8	2.3	35.7	7.8
All trials	9.6	7.3	11.9	7.5	18.7	11.7
